# Early Detection of Dengue Fever Outbreaks Using a Surveillance App (Mozzify): Cross-sectional Mixed Methods Usability Study

**DOI:** 10.2196/19034

**Published:** 2021-03-01

**Authors:** Von Ralph Dane Marquez Herbuela, Tomonori Karita, Thaddeus Marzo Carvajal, Howell Tsai Ho, John Michael Olea Lorena, Rachele Arce Regalado, Girly Dirilo Sobrepeña, Kozo Watanabe

**Affiliations:** 1 Center for Marine Environmental Studies Ehime University Matsuyama Japan; 2 Department of Civil and Environmental Engineering, Graduate School of Science and Engineering Ehime University Matsuyama Japan; 3 Department of Special Needs Education Graduate School of Education Ehime University Matsuyama Japan; 4 Biological Control Research Unit Center for Natural Science and Environmental Research De La Salle University Manila Philippines; 5 College of Arts, Sciences and Education Trinity University of Asia Quezon City Philippines; 6 St. Luke’s College of Nursing Trinity University of Asia Quezon City Philippines; 7 Guidance Counseling and Testing Department University of Santo Tomas–Angelicum College Quezon City Philippines; 8 Pediatrics Department Novaliches District Hospital Quezon City Philippines

**Keywords:** dengue fever, mHealth, public health surveillance, health communication, behavior modification, dengue outbreak

## Abstract

**Background:**

While early detection and effective control of epidemics depend on appropriate surveillance methods, the Philippines bases its dengue fever surveillance system on a passive surveillance method (notifications from barangay/village health centers, municipal or city health offices, hospitals, and clinics). There is no available mHealth (mobile health) app for dengue fever that includes all the appropriate surveillance methods in early detection of disease outbreaks in the country.

**Objective:**

This study aimed to evaluate the usability of the Mozzify app in terms of objective quality (engagement, functionality, aesthetics, information) and app subjective and app-specific qualities and compare total app mean score ratings by sociodemographic profile and self and family dengue fever history to see what factors are associated with high app mean score rating among school-based young adult samples and health care professionals. Individual interviews and focus group discussions were also conducted among participants to develop themes from their comments and suggestions to help structure further improvement and future development of the app.

**Methods:**

User experience sessions were conducted among participants, and the Mobile Application Rating Scale (MARS) professional and user versions (uMARS) were administered followed by individual interviews and focus group discussions. Descriptive statistical analysis of the MARS and uMARS score ratings was performed. The total app mean score ratings by sociodemographic and dengue fever history using nonparametric mean difference analyses were also conducted. Thematic synthesis was used to develop themes from the comments and suggestions raised in individual interviews and focus group discussions.

**Results:**

Mozzify obtained an overall >4 (out of 5) mean score ratings in the MARS and uMARS app objective quality (4.45), subjective (4.17), and specific (4.55) scales among 948 participants (79 health care professionals and 869 school-based samples). Mean difference analyses revealed that total app mean score ratings were not significantly different across ages and gender among health care professionals and across age, income categories, and self and family dengue fever history but not gender (*P*<.001) among the school-based samples. Thematic syntheses revealed 7 major themes: multilanguage options and including other diseases; Android version availability; improvements on the app’s content, design, and engagement; inclusion of users from low-income and rural areas; Wi-Fi connection and app size concerns; data credibility and issues regarding user security and privacy.

**Conclusions:**

With its acceptable performance as perceived by health care professionals and school-based young adults, Mozzify has the potential to be used as a strategic health intervention system for early detection of disease outbreaks in the Philippines. It can be used by health care professionals of any age and gender and by school-based samples of any age, socioeconomic status, and dengue fever history. The study also highlights the feasibility of school-based young adults to use health-related apps for disease prevention.

## Introduction

In 1953, the first dengue hemorrhagic fever outbreak was reported in the Philippines [1]. Since then, this mosquito-borne viral infection that causes acute, potentially severe flu-like illness has been a leading public health burden in all regions of the country [2]. While early detection and effective control of epidemics depend on appropriate surveillance methods [3], the Philippines relies on a passive surveillance method that mainly depends on notifications from barangay/village health centers, municipal or city health offices, hospitals and clinics, and quarantine sections [4-6]. This limits reports of cases that are clinically diagnosed without laboratory confirmation [4,7] which is only a portion (14.3%) of the dengue cases [8,9]. This leaves patients with undifferentiated febrile illness or viral syndrome underreported and, thus, limits the capability to predict or control epidemics [8].

Public health surveillance aims to monitor dengue transmission accurately, triggering the necessary effective preventive measures and programs to prevent the occurrence and spread of diseases [8]. Recently, the use of mHealth (mobile health) technology, specifically mobile apps, has been gaining prominence as a potential surveillance system that would meet the need for real-time disease surveillance and timely identification of epidemics [3,10]. Mohanty et al [3] found 26 apps relevant to epidemic surveillance of diseases such as influenza, H1N1, Ebola, Zika, and dengue. Most of the apps were free of charge and provide real-time tracking and interactive maps, and the majority were on the Android platform only and focus on a single disease (mostly influenza). Some were country and language-specific and had narrow applicability; only a few were tailored to health professionals [3]. Thus, there is a pressing need to develop an app that addresses these limitations.

**Figure 1:**
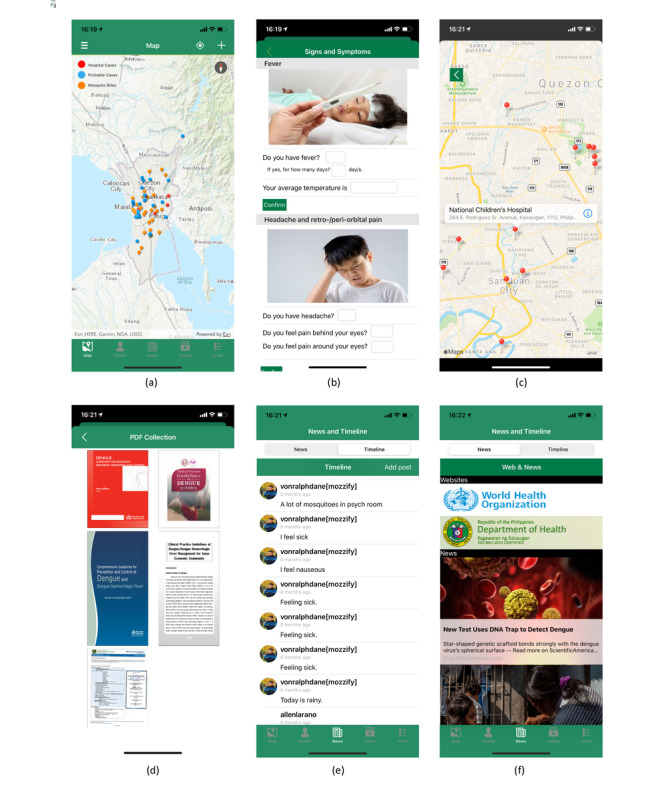
Screenshots of the Mozzify app: (a) Dengue fever cases and mosquito bite reporting and mapping, (b) symptoms checker, (c) hospital directions, (d) within-app local and international PDF collection on Dengue fever, (e) chat forum (timeline), (f) local and international health agency websites and news.

Mozzify is a noncommercial app that features real-time reporting and mapping of dengue cases, comprehensive health communication, and an evidence-based behavior modification system tailored for members of the general public and health care professionals [11] (Figure 1). It is an integrated mHealth app that combines appropriate surveillance methods in the early detection of disease outbreaks: indicator-based surveillance (IBS), event-based surveillance (EBS), and behavior modification [3]. The app includes health care professionals in reporting laboratory-confirmed dengue fever cases, which is the provision of IBS [3,9,11]. It also uses ArcGIS’s spatial analysis feature (Environmental Systems Research Institute) to identify hotspots, which is also an IBS method (sentinel surveillance) [3,11,12]. The reporting and mapping of patients with probable or suspected (with clinical symptoms) dengue fever through its interactive symptoms checker (syndromic surveillance, also an IBS method) is another app feature [3,9,11,13]. Mozzify also includes media reports and news, social media (timeline/chat forum), and links to websites of international and local health agencies to detect and monitor outbreaks which is the provision of the EBS method [3,11,14]. Most importantly, the app promotes behavior modification to address the poor translation of awareness or knowledge of the different preventive practices against dengue fever into practice [11].

Mozzify was pilot tested in Japan and obtained overall mean star ratings of ≥4.0 (out of 5) based on the Mobile Application Rating Scale (MARS) [11] among 5 health experts (4.4), 23 health-related researchers (4.1), and 22 nonclinical end users (4.1). It received ≥4.0 mean score ratings in objective and subjective quality (4.2) and ≥4.4 in specific quality from the participants [11]. The pilot study concluded that Mozzify may be a promising integrated strategic health intervention system for dengue case reporting and mapping, increasing awareness, improving knowledge, and changing attitudes about dengue [11,15]. However, the study had a small sample size and was not conducted in a country where dengue fever is endemic, which limited the generalizability of its findings. Thus, the next step was to evaluate the app’s usability in the Philippines where there were approximately 106,630 dengue fever cases and 456 associated deaths reported from January 1 to June 29, 2019 (morbidity week 1 to week 26), which were higher than the alert and epidemic thresholds [16]. This led to the declarations of a National Dengue Alert on July 15, 2019, followed by a National Dengue Epidemic on August 6, 2019, by the Department of Health (DOH) when the cases reached 271,480, 113% higher than the same period in 2018 [17-20]. To the best of our knowledge, there is no other available integrated mHealth app for dengue fever that includes all the appropriate surveillance methods in early detection of disease outbreaks.

There were approximately 74 million (56.7% of the total population) mobile phone users in the Philippines as of 2019 [21,22]. According to a survey conducted in 2018 among those who own a mobile phone or use the internet (at least occasionally) in the Philippines, 93% are young adults (aged 18 to 29 years), of which 86% are pursuing higher education (those who are in university level and above) [23]. The survey also reported that the second most common activity of mobile phone users in the Philippines is to seek information about health and medicine [23]. Given these data, school-based young adults can be encouraged to use mHealth apps. However, a prior study that involved this population reported that among those who had smartphones, only 14% had mHealth apps [24]. Despite this low use rate, 66.4% of those who had used mHealth apps reported that these apps were useful, and 92.8% were satisfied with the functions of the mHealth apps that they owned [24]. Thus, this population, who comprise 30% of reported cases of dengue fever during the outbreak in 2019, can provide important information on the usability of the Mozzify app in the Philippines [20]. Further, the inclusion of health care professionals who have first-hand experience in the management of patients and are knowledgeable about the disease and its control and prevention will enable the app to address the actual needs of the users for whom it was developed.

Therefore, the objectives of this study were threefold: (1) test and evaluate the usability of the Mozzify app among health care professionals and school-based young adult samples in Metro Manila, Philippines, in terms of engagement, functionality, aesthetics, information, app subjective and app-specific qualities; (2) compare the total app mean score ratings by sociodemographic profile and self and family dengue fever history to identify factors associated with a high app mean score rating; and (3) conduct individual interviews and focus group discussions among the participants and analyze their comments and suggestions to help structure further improvement of the app. This app will be the first to be tested and introduced in the Philippines, specifically Metro Manila during an outbreak. Hence, we hypothesized that the mean score ratings among the participants will be high due to the app’s relevance to the current outbreak and the lack of a surveillance system in the country. We also hypothesized that the total app mean score ratings will not be significantly different across ages, gender, income categories, and self and family dengue fever history, indicating that the app can be used as a potential and appropriate surveillance method in early detection of dengue fever outbreaks.

## Methods

### Study Design and Sampling Method

This cross-sectional mixed-methods usability study was conducted from August to September 2019 in Metro Manila, Philippines, one of the regions that was greatly affected by the dengue fever outbreak with 18,136 reported cases from January 1 to August 31, 2019 [20]. School-based samples were recruited based on inclusion criteria (university students aged between 18 to 29 years who owned a mobile phone) using the respondent-driven sampling technique by group from a university in Quezon City, the largest city with the highest population among the cities in Metro Manila as of 2015 [24,25]. Health care professionals were recruited from academic, clinical, research, or community settings based on the authors’ local networks and through the use of a recruitment poster that was sent or posted via email and social networking sites.

### Ethical Considerations

This study was approved and conducted in accordance with international ethical guidelines (2019-28-Herbuela-VPAA-Mozzify-v1) [26,27]. When found eligible, participants were asked to read and sign (for online Google Forms version, by clicking “Yes, I agree”) an informed consent sheet and were told their participation in the study was voluntary and they may stop their participation at any time. All forms containing personal information were coded and stored in a password-protected database and computer.

### Mozzify App

The app has 3 main components: real-time dengue fever case reporting and mapping system, health communication, and behavior modification. Through the use of ArcGIS, users can report a probable case or mosquito bite incidence by pinning their current location (or any other location). The map counts the pins in each barangay or village and colors them depending on the number of dengue cases. The app lets the user check their symptoms and alerts and navigates to the nearest hospitals that cater to dengue fever patients if it is found they require prompt medical assessment by a physician (determination is made after user answers 26 symptom-related questions) [11]. The app shows different evidence-based local and international guidelines in PDF, videos, and news on the control, prevention, diagnosis, and treatment of dengue fever and websites of the World Health Organization (WHO) and DOH [28,29]. It also has a chat forum (timeline) where users can post events, concerns, and questions about dengue fever. Mozzify promotes behavior modification using an alert system to encourage the practice of preventive measures against dengue based on WHO’s communication for behavioral impact strategy [15,30,31].

### Usability Testing

**Figure 2:**
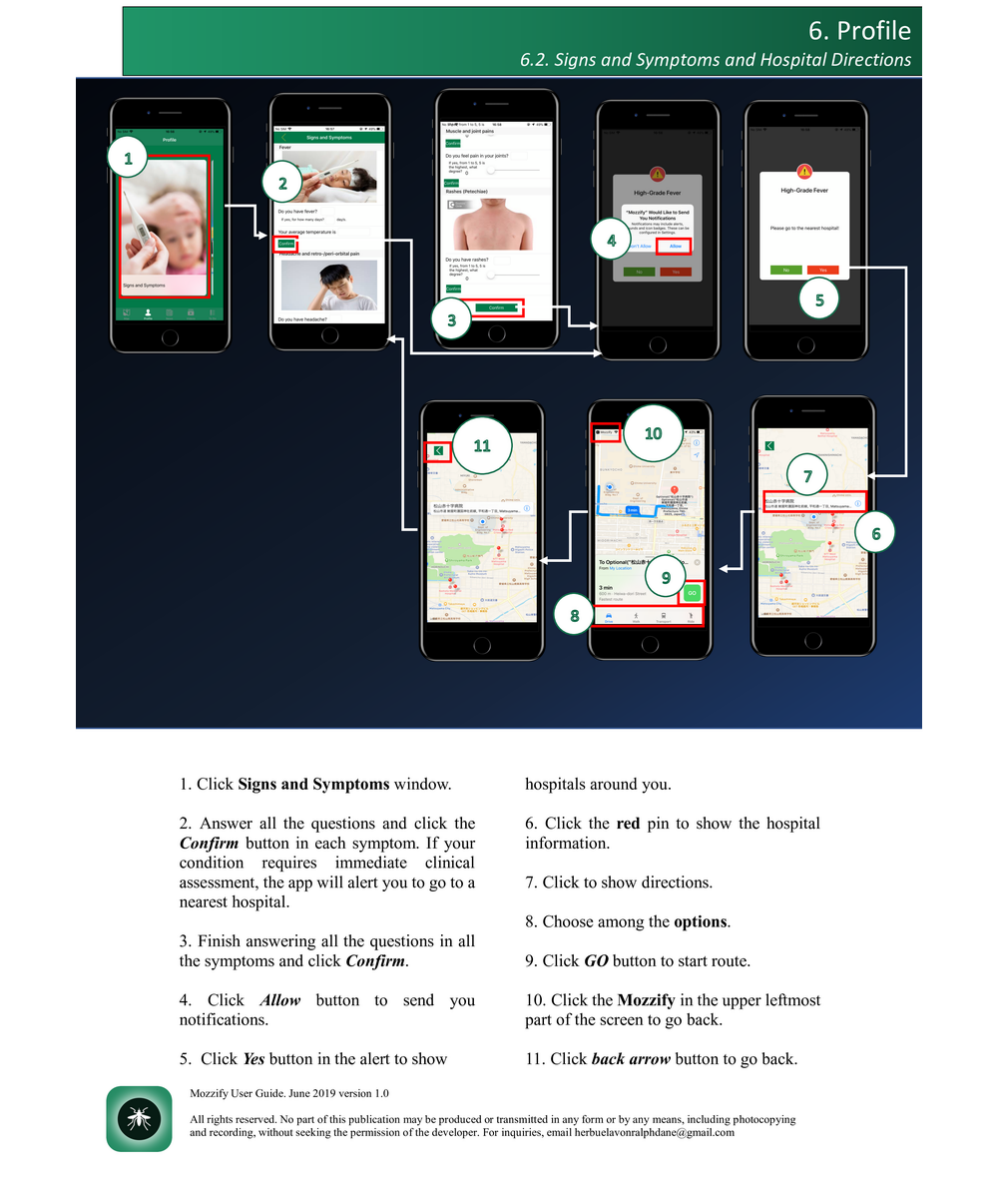
Sample screenshot of the app user guide.

Usability testing was done to investigate the user-Mozzify interaction in terms of its functions, engagement, design, information, subjective and specific qualities, effectiveness, efficiency, and satisfaction of the users to produce recommendations for the design (or redesign), structure, or further iterations [32-35]. All participants were asked to install the app on their mobile phones; otherwise, they were asked to use the available mobile phones with the installed Mozzify app. The majority of health care professionals were invited for the individual user experience sessions, while school-based samples were invited for a series of 1-hour investigator-facilitated group user experience sessions. In the first 15 minutes, the investigator used the screen projection method to show the mobile app and its functions to the participants and let them perform the tasks based on a predetermined checklist matched with the functions of the app. However, in cases when participants could not attend the sessions, they were asked to use the app’s user guide (Figure 2) or explore the app as they would do in other apps.

### Measures of Interest and Outcomes

#### Sociodemographic Profile

Participants were asked to provide their sociodemographic information such as age and gender to allow investigation of mean score differences within its categories. Age was clustered according to the minimum and maximum (aged 18 to 19 years for school-based samples and 19 to 53 years for health care professionals) ages of the participants with an equal interval size of 4 (eg, 18-21, 22-25). The school-based samples were asked to provide their family monthly income, which was categorized (adjusted to 10,000-peso interval size) using the clusters from the indicative range of monthly family incomes (for a family of 5) based on a study on the profile and determinants of the middle-income class in the Philippines in 2015 and 2017 [36]. Information on self and family history of dengue fever was collected from school-based samples to investigate whether a previous diagnosis or history of dengue fever in a family would be associated with higher total mean score app ratings.

#### MARS

The second phase of testing included the use of a standardized self-administered questionnaire (Multimedia Appendix 1). Two versions of the MARS, the professional 23-item MARS and the 20-item user version (uMARS), were used to evaluate the usability of Mozzify among health care professionals and the school-based samples either online or on paper [32,37]. Both versions use a 5-point scale (1=inadequate to 5=excellent) to assess the app in terms of objective quality (engagement, functionality, aesthetics, and information), subjective quality (4 items on recommending the app to others, using the app for a short or long time, and overall star rating of the app), and the app-specific quality (perceived impact of the app on the user’s awareness and knowledge of and attitudes toward dengue fever, help-seeking behavior, intentions, and actual change of behavior in practicing preventive measures) [32,37]. Both versions had excellent internal consistency level Cronbach alpha=.90 [32,37].

#### Individual Interviews and Focus Group Discussions

After answering the questionnaire, health care professionals were invited to individual interviews and school-based samples invited to focus group discussions to provide more comprehensive qualitative feedback. The sessions were completed by one trained investigator who was guided with preselected questions (semistructured), and all the participants were asked to write their comments and suggestions to ensure consistency in the analysis.

### Statistical and Data Analysis

Statistical analysis was conducted using SPSS Statistics version 25 (IBM Corporation). All items on the MARS and uMARS were scored based on responses on the 5-point Likert scale (5 points=excellent, 4 points=good, etc) [37]. The mean score of each subscale in the app objective quality scale, each item of the app subjective quality scale (reported as individual items and by mean score), and the app-specific scale were calculated separately for the school-based samples and health care professionals. The mean scores of each scale were then combined to get the overall mean score among the participants, which was also used to investigate the differences in means within each category of age, gender, income, and dengue fever history (self and family). Nonparametric Mann-Whitney *U* tests and Kruskal-Wallis tests were used for dichotomous and multicategorical variables, respectively, as normality tests revealed that the data were not normally distributed (*P*<.001).

Comments and suggestions in the individual interviews and group discussions were analyzed using thematic synthesis [38]. The transcripts of the comments and suggestions of the participants were encoded verbatim to an Excel file (Microsoft Corporation) as a database. Topic and key concept or content unit was identified by examining (word-by-word) each transcript [39]. The identified content units were used to easily add to a bank of codes for themes, and new ones were developed as required. Similar or overlapping content units were coded (with numbers that corresponded to a theme) and quantified using quantitative content analysis where each content unit was scored 1 [40]. The similar or overlapping content units were grouped into minor themes and then grouped to major themes. The frequencies of content units in each theme were added to identify its relevance.

## Results

### Participant and Data Flow

**Figure 3:**
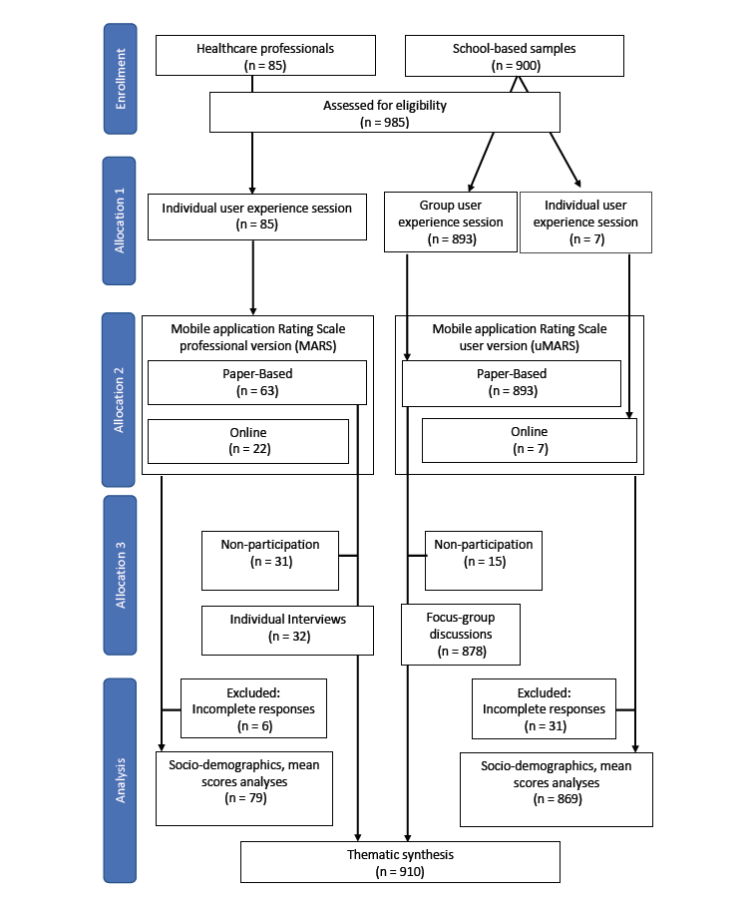
Consolidated Standards of Reporting Trials diagram of participant flow from enrollment, allocation, and analysis.

We were able to recruit 985 eligible participants (health care professionals, n=85; school-based young adult samples, n=900). All of the health care professionals and school-based samples attended the user experience sessions. Of the health care professionals, 37.6% (32/85) were able to attend the individual interviews, while 98.3% (878/893) of school-based samples attended the focus group discussions. The Consolidated Standards of Reporting Trials (CONSORT) diagram (Figure 3) shows the detailed participant flow from user experience sessions, excluding (incomplete) responses, to the number of responses included in the analysis.

### Sociodemographic and Dengue Fever History Profile

The school-based samples were mostly female (549/869, 63.2%) aged 18 to 21 years (698/869, 89.6%) with a mean age of 19.5 years; 28.8% (131/455) come from a family with a monthly income of ≥50,000 pesos. Three-quarters (417/555, 75.1%) had no dengue fever history, and 55.7% (306/550) had no family members with a history of dengue fever, as shown in Table 1. Health care professionals had a mean age of 31.8 years with a nearly equal distribution by gender (35/79, 50.7% females).

**Table 1:** Sociodemographic and dengue fever history profile of health care professionals and school-based samples.

Sociodemographic profile		Health care professionals (n=79), n (%)	School-based samples (n=869), n (%)
**Gender**			
	Male	35 (49.3)	234 (26.9)
	Female	36 (50.7)	549 (63.2)
**Age in years**			
	18-21	3 (5.17)	698 (89.6)
	22-25	14 (24.1)	68 (8.73)
	26-29	12 (20.7)	13 (1.67)
	30-33	12 (20.7)	—^a^
	34-37	7 (12.1)	—
	38-41	4 (6.90)	—
	42-45	4 (6.90)	—
	46-49	3 (5.17)	—
	50-53	2 (3.45)	—
**Income ( 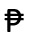 ^b^)**			
	≤10,000	—	46 (10.1)
	11,000-20,000	—	80 (17.6)
	21,000-30,000	—	90 (19.8)
	31,000-40,000	—	58 (12.8)
	41,000-50,000	—	50 (11.01)
	≥51,000	—	131 (28.8)
**Self DF^c^ history**			
	None	—	417 (75.3)
	Had DF	—	138 (24.9)
**Family DF history**			
	None	—	306 (55.7)
	≥1 member had DF	—	244 (44.4)

^a^N/A: not applicable.

^b^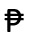: Philippine peso (US $1=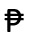
50.5; 5-year average rate).

^c^DF: dengue fever.

### App Objective, Subjective, and Specific Quality

Mozzify obtained >4 (out of 5) mean score ratings in all app objective quality scales among the participants as shown in Table 2. Mean score ratings of >4.50 were found in the information subscale (mean 4.56 [SD 0.6]) among school-based young adult samples, followed by functionality (mean 4.26 [SD 0.51]) and engagement (mean 4.26 [SD 0.45]) among the health care professionals. In the app subjective scale, the item recommending the app to others obtained the highest mean score ratings of 4.58 (SD 0.70) among the school-based sample and 4.62 (SD 0.63) with the health care professionals. In the app-specific scale, improving awareness, knowledge, and help-seeking behavior were the items with the highest mean score ratings (4.59, 4.52, and 4.59, respectively) among health care professionals while improving awareness and help-seeking behavior items got the highest mean score ratings of 4.63 and 4.61, respectively among school-based samples. Conversely, the item on using the app for the next 12 months under app subjective quality saw the lowest mean score ratings of 3.64.

**Table 2:** Mean scores of app objective, subjective, and specific quality ratings based on the Mobile Application Rating Scale from health care professionals and school-based samples.

MARS^a^/uMARS^b^ subscales		Health care professionals (n=79), mean (SD)		School-based samples (n=869), mean (SD)		Combined (n=948), mean (SD)
**App objective quality**		4.22 (0.59)		4.47 (0.69)		4.45 (0.67)
	1. Engagement	4.26 (0.45)	4.39 (0.75)		4.38 (0.74)	
	2. Functionality	4.26 (0.51)	4.46 (0.69)		4.45 (0.68)	
	3. Aesthetics	4.17 (0.49)	4.47 (0.67)		4.44 (0.67)	
	4. Information	4.19 (0.62)	4.56 (0.63)		4.53 (0.64)	
**App subjective quality**		3.71 (0.57)		4.17 (0.88)		4.13 (0.88)
	5. Recommending the app to others	4.62 (0.63)	4.58 (0.70)		4.59 (0.69)	
	6. Using the app for the next 12 months	3.35 (0.79)	3.67 (0.92)		3.64 (0.91)	
	7. Overall (star) rating of the app	4.42 (0.67)	4.25 (0.74)		4.27 (0.73)	
**App specific quality**		4.48 (0.52)		4.55 (0.67)		4.54 (0.67)
	8. Awareness (DF^c^ symptoms, hospital locations, hotspots, prevention, treatment)	4.59 (0.63)	4.61 (0.66)		4.61 (0.66)	
	9. Knowledge (DF symptoms, prevention, treatment)	4.52 (0.64)	4.58 (0.66)		4.58 (0.66)	
	10. Attitude (severity, susceptibility, preventive practices against DF)	4.37 (0.64)	4.42 (0.72)		4.41 (0.72)	
	11. Intention-to-change (preventive practices against DF)	4.43 (0.61)	4.49 (0.67)		4.48 (0.67)	
	12. Help-seeking (for clinical assessment for presence of DF symptoms)	4.59 (0.63)	4.63 (0.63)		4.63 (0.63)	
	13. Behavior change (preventive practices against DF)	4.35 (0.56)	4.57 (0.67)		4.55 (0.66)	

^a^MARS: Mobile Application Rating Scale.

^b^uMARS: Mobile Application Rating Scale–User Version.

^c^DF: dengue fever.

### Mean Score Differences by Sociodemographics and Dengue Fever History

App mean score ratings were not significantly different across age and gender among health care professionals and age, income brackets, and self and family dengue fever history, except gender among school-based samples as shown in Table 3. Females had significantly (*P*<.001) higher app mean score ratings (mean 4.50 [SD 0.41]) than males (mean 4.34 [SD 0.52]).

**Table 3:** Total mean score comparison among health care professionals and school-based samples by sociodemographic and dengue fever history.

Sociodemographic profile			Health care professionals (n=79)			School-based samples (n=869)		
			Mean (SD)	*P* value		Mean (SD)		*P* value
**Gender^a^**								
	Male	4.31 (0.32)		.34	4.34 (0.52)		<.001	
	Female	4.22 (0.40)		—^b^	4.50 (0.41)		—	
**Age in years^c^**								
	18-21	4.69 (0.02)		.67	4.45 (0.46)		.97	
	22-25	4.23 (0.18)		—	4.47 (0.42)		—	
	26-29	4.39 (0.40)		—	4.43 (0.52)		—	
	30-33	4.26 (0.34)		—	—		—	
	34-37	4.40 (0.40)		—	—		—	
	38-41	4.16 (0.74)		—	—		—	
	42-45	4.35 (0.27)		—	—		—	
	46-49	4.46 (0.48)		—	—		—	
	50-53	4.19 (0.08)		—	—		—	
**Income ( 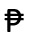 ^d^)^c^**								
	≤10,000	—		—	4.47 (0.58)		.18	
	11,000-20,000	—		—	4.45 (0.41)		—	
	21,000-30,000	—		—	4.42 (0.50)		—	
	31,000-40,000	—		—	4.38 (0.54)		—	
	41,000-50,000	—		—	4.33 (0.42)		—	
	≥51,000	—		—	4.48 (0.39)		—	
**Self DF^e^ history^b,c^**								
	None	—		—	4.46 (0.44)		.61	
	Had DF	—		—	4.42 (0.48)		—	
**Family DF history^b,c^**								
	None	—		—	4.44 (0.46)		.29	
	≥1 member had DF	—		—	4.47 (0.43)		—	

^a^Mann-Whitney *U* test.

^b^N/A: not applicable.

^c^Kruskal-Wallis test.

^d^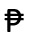: Philippine peso (US $1=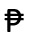
50.5; 5-year average rate).

^e^DF: dengue fever.

### Individual Interviews and Focus Group Discussions

#### Thematic Synthesis

Of the 910 attendees (32 health care professionals and 878 school-based samples), only 424 transcripts were collected from 26 (out of 32) health care professionals and 398 (out of 878) school-based young adult samples. From 424 transcripts analyzed, a total of 443 content units (47 from the health care professionals and 369 from the school-based samples) were identified and grouped into 16 minor themes that were then grouped into 7 major themes as shown in Table 4: positive comments regarding the app’s concept, design, information, and features (320/443, 72.2%); suggestions on adding features like multilanguage options and including other diseases (48/443, 10.8%); Android version availability (36/443, 8.13%); inclusion of users from low-income and rural areas (14/443, 3.16%); improvements on the app’s content, design, and engagement (14/443, 3.16%); Wi-Fi connection and app size concerns (6/443, 1.35%); and data credibility and issues regarding user security and privacy (5/443, 1.13%).

**Table 4:** The major themes that emerged from individual interviews and focus group discussions among health care professionals and school-based samples.

Themes	Content units		
	Health care professionals (n=47)	School-based samples (n=396)	Combined (n=443)
1. Positive comments (concept, design, information, features)	27 (57.4)	293 (73.9)	320 (72.2)
2. Suggestions (add features: multilanguage, include other diseases, alerts)	11 (23.4)	37 (9.34)	48 (10.8)
3. Android version availability	6 (12.8)	30 (7.58)	36 (8.13)
4. Inclusivity (rural areas, low-income families)	2 (4.33)	12 (3.03)	14 (3.16)
5. Improvement (content and design, engagement)	—^a^	14 (3.54)	14 (3.16)
6. Wi-Fi connection and app size	—	6 (1.52)	6 (1.35)
7. Data credibility and user security and privacy	1 (2.1)	4 (1.01)	5 (1.13)

^a^N/A: not available.

#### Positive Comments

Almost three quarters (320/443, 72.2%) of the topics discussed in individual and group discussions included positive comments regarding the app. Health care professionals perceived that the app is concise and relevant, interesting, well-executed, and informative. They also perceived that the app will be of help to clinicians in improving health care awareness and services to the people, especially those patients with dengue fever. The school-based samples also had positive comments about the app: user-friendly, useful, educational, innovative, well-designed, interactive, and unique. Some commented about the capability of the app to improve public awareness of dengue fever.

This mobile app is what we need today to raise medical awareness about vector-borne diseases. This is timely because the country is facing a big dilemma on dengue. This could help doctors in locating possible dengue cases so that proper medical attention will be given. Looking forward to using this app and hoping to be approved by the Department of Health because it really helps in our country to give awareness and knowledge about dengue, and in future the high increase of dengue cases will be decreased.

#### Suggestions

Participants also suggested including some additional features and information that they perceived should be in the app. Suggestions including adding a feature that would allow users to easily access medical services professionals, multilanguage options for users, more information (through videos, graphics, and PDF files) about vector mosquitoes, information about the dengue vaccine, how to prevent or avoid dengue, vector-human interaction, and including other diseases (eg, HIV, tuberculosis, other mosquito-borne diseases). Participants also suggested connecting the app to social media like Facebook and Twitter, adding advertisements and games, and developing a desktop version for hospitals and clinics.

Maybe link, a feature that can immediately notify the nearby hospital for assistance; add emergency hotlines of nearby hospitals/clinics. Have multiple languages, so a particular country can use their own languages to better understand the use of the app. This app should also include other diseases caused by mosquitoes. Maybe this app can also be applied not only for dengue cases or human health–related but also for environmental issues and disease outbreaks in the agriculture sector. Include videos or mini games that would make the app more interactive.

#### Android Version

A total of 36 (36/443, 8.13%) topics were raised regarding the need for an Android version of the app.

The app should be available to all and not restricted to iOS to fully utilize its intention to help raise awareness about these diseases caused by mosquitoes.

#### Inclusivity

Another major theme is the need for inclusivity (14/443, 3.02%). Participants commented that the app should bridge the gap between low-income and those who are in rural areas and receiving appropriate medical services through the use of the app.

This is a novel approach to mitigate the barriers between rural areas and health care. The people living in the line of poverty might not be able to access the app immediately and since most people who are experiencing poverty are the ones most vulnerable to dengue; Needs to be more accessible to people in different social classes.

#### Improvement

This theme emerged based on the comments of members of the school-based sample regarding improving the app’s design, color, and mapping system and making the app more interactive (14/396, 3.54%).

Enhance the graphics to be more aesthetically pleasing so more would be attracted to use it often. It should be more colorful to attract millennial students or even kids/teens. The user interface seems pretty bare, so maybe make it a bit more appealing.

#### Wi-Fi Connection and App Size

Concerns regarding limited access to Wi-Fi connections and the huge size of the app (6/396, 1.52%) were raised by the members of the school-based samples.

It is a useful app but what about for people who don’t have strong Wi-Fi connections. I am not sure either if the PDFs and videos that supposedly can play without internet are required since I assume this will take a lot of space within the phone’s storage.

#### Data Credibility and Issues Regarding User Security and Privacy

The last but probably one of the most important themes emerging were concerns regarding data security and privacy issues (5/443, 1.13%).

How can we prevent the abuse of misinformation and protect the credibility of the app? Concern regarding validation and data privacy act. Accessibility with all which is not advisable due to discrimination.

## Discussion

### Principal Findings

Mozzify obtained high mean score ratings (>4 out of 5) in the MARS app objective, subjective, and specific quality scales. Mean difference analyses revealed that total app mean score ratings were not significantly different across ages among health care professionals and across income categories and self and family dengue fever history but not gender among school-based samples. Thematic synthesis of the topics discussed in the individual interviews and focus group discussions revealed 7 major themes.

This study extends the pilot study conducted in Japan by including a significantly large sample size in the Philippines where dengue fever is endemic [14]. To our knowledge, Mozzify is the first mobile app for dengue fever that has been tested and introduced in the Philippines, especially in Metro Manila. Our study also provides the first report on the perception of health care professionals and school-based samples regarding the app’s usability in terms of its functions, engagement, design, information, and subjective and specific qualities.

The high mean score ratings (>4 out of 5) in the MARS app objective quality scale, specifically in the information and functionality subscales, indicate that it contains high-quality information from a credible source and excellent functioning, easy-to-learn navigation flow, logic, and gestural design. The high mean score ratings of the item recommending the app to others in the subjective quality scale indicate that the participants had a high desire or intention to recommend the app to others. Most importantly, the results in the app-specific scale indicate that the app has the potential to improve user awareness and knowledge of dengue fever symptoms, hotspots, prevention, and management; encourage help-seeking behavior among users through its unique and interactive symptoms checker and hospital navigation system; improve users’ intentions; and encourage users to develop or practice preventive measures against dengue fever.

The total app mean score ratings, which were not significantly different across ages, indicate that Mozzify is relevant and can be used as a potential surveillance method for dengue fever as perceived by health care professionals and school-based samples of any age (above 18 years). This perception is also true among school-based samples of any socioeconomic status with or without a self or family history of dengue fever. Surprisingly, among the school-based samples, females had a significantly (*P*<.001) higher total app mean score rating than males. Aside from the fact that there were more females (549/869, 63.2%) than males (234/869, 26.9%) among the school-based samples, the nearly equal distribution of males and females whose mean ratings were not significantly different (*P*=.34) among health care professionals suggests that the app mean score ratings between female and male school-based samples would not also be statistically different if they would have had equal sample sizes.

The interview and discussion sessions revealed that while the participants had positive perceptions of the app, they also suggested adding features like multilanguage options and including other diseases. As mentioned earlier, most of the available mHealth apps for epidemic surveillance focus on a single disease (mostly influenza), and only a few were tailored for health professionals [3]. While the latter has been included from conception to evaluation of Mozzify, including other mosquito-borne diseases (eg, malaria, Zika, chikungunya, and Japanese encephalitis) in the app has been considered, hence the name Mozzify, which was termed after the word mosquito. To our knowledge, one similar app called FeverDX has included different vector-borne diseases and, like Mozzify, has received a high mean score rating of 4 (out of 5) among health care professionals [41].

Enhancing the app’s engagement ability also emerged as one of the concerns of the participants. The relatively low mean score rating on using the app in the next 12 months can be associated with the relatively low overall mean score rating of 4.38 in the engagement subscale under the app objective quality scale. These emphasize the need to sustain the engagement of the users with the app for longer use and the possibility of including games, a potential tool to increase engagement among users that can be integrated with health behavior change concepts [42]. Moreover, the participants also suggested that the app should have an Android version and an added alert system. While some of the epidemic surveillance apps run on the Android platform [3], Mozzify is only available in iOS. An Android version has been developed, and an alert system has been designed in the map that would warn users when they enter or when they are near a barangay/village or area with high dengue fever case incidence and mosquito abundance. Currently, a version with multilanguage options is being developed in preparation for its use in other countries where dengue is prevalent.

The interview and discussion sessions also introduced important factors that should be considered in the further improvement of the app. First, a stable Wi-Fi connection is indeed necessary for most of the features of the app to function. ArcGIS has an offline version of the online map; however, it does not allow users to input mosquito bite reports and dengue fever cases. This has been addressed by directly installing the videos and PDFs of online-sourced evidence-based local and international guidelines in the app. However, it increases the app’s size which is another concern. Second, and probably the most important, are issues regarding the credibility of user data inputs and security and privacy. To ensure the credibility of data inputs from users, a mechanism to include email or phone number verification among users when signing up for the app that would allow the system to track suspicious accounts has been designed. Also, a plan to connect with and train health care workers to confirm inputted data or users within their barangays or neighborhood has been in place. However, there is also a need to limit the access of the online map that shows cases among health care providers and local government officials to aid in proper planning and mitigation with the affected areas.

### Limitations

This study has several limitations. First, participants resided in urbanized areas and thus, the findings may not represent those who are in rural areas. Second, participants only used and assessed the app in a very short span of time, limiting the evaluation to the app’s design and usability. Third, the investigator-led user experience sessions conducted may bias the results of this study as they may not reflect a real-world scenario. Fourth, the experiences and perceptions of the school-based young adult samples in this study may not represent that of the majority of young adults. Fifth, the participants were mostly students and health care professionals in an academic and research-based setting, which could confound their true assessment of the app since they are more involved in academic- or research-approach assessment and may be more inclined to give positive responses. These limitations should be addressed by conducting a longitudinal (eg, 8-month use of the app) implementation study in urban and rural communities that may give insights on the app’s actual use in a real-world scenario (eg, number of reported cases, pre and post levels of perceived change in awareness, knowledge on, and attitudes toward dengue fever, help-seeking behavior, intentions, and actual change of behavior in practicing preventive measures against dengue fever).

### Conclusions

Despite these limitations, Mozzify, with its acceptable performance as perceived by health care professionals and school-based young adults, has the potential to be used as a strategic health intervention system in early detection of disease outbreaks in the Philippines. Based on our results, it could also be used by health care professionals and school-based samples of any age group, gender, and socioeconomic status. The study also emphasizes the participation of young adults in the usability testing of Mozzify, which highlights the feasibility of this population to use health-related apps for disease prevention.

## Appendix

Multimedia Appendix 1 Mobile Application Rating Scale form.

## Abbreviations

CONSORT: Consolidated Standards of Reporting Trials
DOH: Department of Health
EBS: event-based surveillance
IBS: indicator-based surveillance
JSPS: Japan Society for the Promotion of Science
MARS: Mobile Application Rating Scale
mHealth: mobile health
uMARS: Mobile Application Rating Scale–User Version
WHO: World Health Organization

## References

[1] Gubler DJ (1998). Dengue and dengue hemorrhagic fever. Clin Microbiol Rev.

[2] (2018). Dengue and severe dengue. World Health Organization.

[3] Mohanty B, Chughtai A, Rabhi F (2019). Use of mobile apps for epidemic surveillance and response: availability and gaps. Global Biosecurity.

[4] Edillo FE, Halasa YA, Largo FM, Erasmo JNV, Amoin NB, Alera MTP, Yoon I, Alcantara AC, Shepard DS (2015). Economic cost and burden of dengue in the Philippines. Am J Trop Med Hyg.

[5] Edillo F, Madarieta S (2012). Trends of dengue infections (1997-2008) in Cebu Province, Philippines. Dengue Bull.

[6] National Epidemiology Center of the Department of Health (2008). Manual of Procedures for the Philippine Integrated Disease Surveillance and Response. 1st Edition.

[7] Shepard DS, Undurraga EA, Halasa YA (2013). Economic and disease burden of dengue in Southeast Asia. PLoS Negl Trop Dis.

[8] Ooi E, Gubler DJ (2009). Dengue in Southeast Asia: epidemiological characteristics and strategic challenges in disease prevention. Cad Saude Publica.

[9] Yan SJ, Chughtai AA, Macintyre CR (2017). Utility and potential of rapid epidemic intelligence from internet-based sources. Int J Infect Dis.

[10] El-Khatib Z, Shah M, Zallappa SN, Nabeth P, Guerra J, Manengu CT, Yao M, Philibert A, Massina L, Staiger C, Mbailao R, Kouli J, Mboma H, Duc G, Inagbe D, Barry AB, Dumont T, Cavailler P, Quere M, Willett B, Reaiche S, de Ribaucourt H, Reeder B (2018). SMS-based smartphone application for disease surveillance has doubled completeness and timeliness in a limited-resource setting: evaluation of a 15-week pilot program in Central African Republic (CAR). Confl Health.

[11] Herbuela VRDM, Karita T, Francisco ME, Watanabe K (2020). An integrated mHealth app for dengue reporting and mapping, health communication, and behavior modification: development and assessment of Mozzify. JMIR Form Res.

[12] The National Center for Biotechnology Information (NCBI) MeSH Database: Sentinel Surveillance. The National Center for Biotechnology Information (NCBI).

[13] Flamand C, Quenel P, Ardillon V, Carvalho L, Bringay S, Teisseire M (2011). The epidemiologic surveillance of dengue-fever in French Guiana: when achievements trigger higher goals. Stud Health Technol Inform.

[14] Global Health Protection and Security. Centers For Disease Control and Prevention.

[15] Herbuela VRDM, de Guzman FS, Sobrepeña GD, Claudio ABF, Tomas ACV, Arriola-Delos Reyes CM, Regalado RA, Teodoro MM, Watanabe K (2019). Knowledge, attitude, and practices regarding dengue fever among pediatric and adult in-patients in Metro Manila, Philippines. Int J Environ Res Public Health.

[16] Department of Health (2019). Monthly Dengue Cases Report No 6, January 1 to June 29, 2019. Epidemiology Bureau, Public Health Surveillance Division.

[17] (2019). Situation report 1: dengue outbreak. Representative Office for the Philippines, World Health Organization.

[18] (2019). Situation report 4: dengue outbreak. Representative Office for the Philippines, World Health Organization.

[19] (2019). Situation report 6. Representative Office for the Philippines, World Health Organization.

[20] (2019). Monthly dengue cases report No. 8, January 1 to August 31, 2019. Epidemiology Bureau, Public Health Surveillance Division.

[21] Sanchez M Smartphone penetration as share of population Philippines 2017-2025. Statista.

[22] Sanchez M (2020). Smartphone users in the Philippines 2015-2025. Statista.

[23] Silver L, Huang C (2019). Emerging economies, smartphone and social media users have broader social networks.

[24] Do TTT, Le MD, Van Nguyen T, Tran BX, Le HT, Nguyen HD, Nguyen LH, Nguyen CT, Tran TD, Latkin CA, Ho RCM, Zhang MWB (2018). Receptiveness and preferences of health-related smartphone applications among Vietnamese youth and young adults. BMC Public Health.

[25] National Capital Region 2015 Census of Population. Philippine Statistics Office.

[26] World Medical Association (2013). World Medical Association Declaration of Helsinki: ethical principles for medical research involving human subjects. JAMA.

[27] European Medicines Agency ICH Topic E6 (R1) Guideline for good clinical practice step 5 note for guidance on good clinical practice (CPMP/ICH/135/95).

[28] World Health Organization (2020). Fact sheets: dengue and severe dengue.

[29] Search results: dengue. Department of Health.

[30] Kumaran E, Doum D, Keo V, Sokha L, Sam B, Chan V, Alexander N, Bradley J, Liverani M, Prasetyo DB, Rachmat A, Lopes S, Hii J, Rithea L, Shafique M, Hustedt J (2018). Dengue knowledge, attitudes and practices and their impact on community-based vector control in rural Cambodia. PLoS Negl Trop Dis.

[31] Parks W, Lloyd L (2004). Planning social mobilization and communication for dengue fever prevention and control: a step-by-step guide.

[32] Stoyanov SR, Hides L, Kavanagh DJ, Zelenko O, Tjondronegoro D, Mani M (2015). Mobile app rating scale: a new tool for assessing the quality of health mobile apps. JMIR Mhealth Uhealth.

[33] Delikostidis I (2007). Methods and Techniques for Field-Based Usability Testing of Mobile Geo-Applications [Thesis].

[34] Khan S, Timmings C, Moore JE, Marquez C, Pyka K, Gheihman G, Straus SE (2014). The development of an online decision support tool for organizational readiness for change. Implementation Sci.

[35] Maramba I, Chatterjee A, Newman C (2019). Methods of usability testing in the development of eHealth applications: a scoping review. Int J Med Inf.

[36] Albert J, Santos A, Vizmanos J (2018). Profile and determinants of the middle-income class in the Philippines.

[37] Stoyanov SR, Hides L, Kavanagh DJ, Wilson H (2016). Development and validation of the user version of the Mobile Application Rating Scale (uMARS). JMIR Mhealth Uhealth.

[38] Urquhart C (2010). Systematic reviewing, meta-analysis and meta-synthesis for evidence-based library and information science.

[39] Campbell R, Pound P, Pope C, Britten N, Pill R, Morgan M, Donovan J (2003). Evaluating meta-ethnography: a synthesis of qualitative research on lay experiences of diabetes and diabetes care. Soc Sci Med.

[40] Morgan DL (1993). Qualitative content analysis: a guide to paths not taken. Qual Health Res.

[41] Rodríguez S, Sanz AM, Llano G, Navarro A, Parra-Lara LG, Krystosik AR, Rosso F (2020). Acceptability and usability of a mobile application for management and surveillance of vector-borne diseases in Colombia: an implementation study. PLoS One.

[42] Cugelman B (2013). Gamification: what it is and why it matters to digital health behavior change developers. JMIR Serious Games.

