# Correction: Early Detection of Dengue Fever Outbreaks Using a Surveillance App (Mozzify): Cross-sectional Mixed Methods Usability Study

**DOI:** 10.2196/29795

**Published:** 2021-04-22

**Authors:** Von Ralph Dane Marquez Herbuela, Tomonori Karita, Thaddeus Marzo Carvajal, Howell Tsai Ho, John Michael Olea Lorena, Rachele Arce Regalado, Girly Dirilo Sobrepeña, Kozo Watanabe

**Affiliations:** 1 Center for Marine Environmental Studies Ehime University Matsuyama Japan; 2 Department of Civil and Environmental Engineering, Graduate School of Science and Engineering Ehime University Matsuyama Japan; 3 Department of Special Needs Education Graduate School of Education Ehime University Matsuyama Japan; 4 Biological Control Research Unit Center for Natural Science and Environmental Research De La Salle University Manila Philippines; 5 College of Arts, Sciences and Education Trinity University of Asia Quezon City Philippines; 6 St. Luke’s College of Nursing Trinity University of Asia Quezon City Philippines; 7 Guidance Counseling and Testing Department University of Santo Tomas–Angelicum College Quezon City Philippines; 8 Pediatrics Department Novaliches District Hospital Quezon City Philippines

In “Early Detection of Dengue Fever Outbreaks Using a Surveillance App (Mozzify): Cross-sectional Mixed Methods Usability Study” (JMIR Public Health Surveill 2021;7(3):e19034) two errors were noted.

In the originally published manuscript, the footnotes of Tables 1 and 3 listed incorrect currency conversions. In Table 1, footnote “b” originally read:

Philippine peso (US $48.10=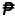
1)

In Table 3, footnote “d” originally read:

Philippine peso (US $52.16=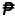
1)

In the corrected version of the manuscript, both of these footnotes have been changed to read:

Philippine peso (US $1=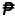
50.5; 5-year average rate)

The correction will appear in the online version of the paper on the JMIR Publications website on April 22, 2021, together with the publication of this correction notice. Because this was made after submission to PubMed, PubMed Central, and other full-text repositories, the corrected article has also been resubmitted to those repositories.

